# Single-Step Engineered Gelatin-Based Hydrogel for Integrated Prevention of Postoperative Adhesion and Promotion of Wound Healing

**DOI:** 10.3390/gels11100797

**Published:** 2025-10-02

**Authors:** Xinyu Wu, Lei Sun, Jianmei Chen, Meiling Su, Zongguang Liu

**Affiliations:** 1 Key Laboratory of the Jiangsu Higher Education Institutions for Integrated Traditional Chinese and Western Medicine in Senile Diseases Control, School of Traditional Chinese Medicine, Faculty of Medicine, Yangzhou University, Yangzhou 225009, Chinacjm@yzu.edu.cn (J.C.);; 2 School of Pharmacy, East China Normal University, Shanghai 200062, China; ylyh_sl@163.com; 3 Microelectronics Industry Research Institute, College of Physics Science and Technology, Yangzhou University, Yangzhou 225009, China

**Keywords:** gelatin, tea polyphenol, antioxidant, wound healing, anti-postoperative adhesion

## Abstract

Postoperative adhesion remains a major clinical challenge, often leading to chronic pain, functional disorders, and recurrent surgeries. Herein, we developed a multifunctional gelatin–polyphenol hydrogel (GPP20) featuring rapid gelation (within 5 min), strong tissue adhesion (lasting > 24 h under physiological conditions), and intrinsic wound healing capacity to achieve integrated prevention of postoperative adhesion. GPP20 was fabricated via dynamic crosslinking between gelatin and tea polyphenol, endowing it with injectability, self-healing, biodegradability, and excellent mechanical properties (shear stress of 14.2 N). In vitro studies demonstrated that GPP20 exhibited effective ROS scavenging (82% ABTS scavenging capability), which protects cells against oxidative stress, while possessing excellent hemocompatibility and in vivo safety. Notably, GPP20 significantly reduced postoperative cecum–abdominal wall adhesions through both physical barrier effects and modulation of inflammation and collagen deposition, demonstrating a comprehensive integrated prevention strategy. Furthermore, in full-thickness wound models, GPP20 accelerated tissue regeneration (85% wound closure rate on day 10) by promoting macrophage polarization toward the M2 phenotype and stimulating angiogenesis, thereby enhancing collagen deposition and re-epithelialization. Collectively, these findings demonstrate that GPP20 integrates anti-adhesion efficacy with regenerative support, offering a facile and clinically translatable strategy for postoperative care and wound healing.

## 1. Introduction

Postoperative adhesion refers to aberrant fibrous connections that develop during healing after abdominal or pelvic surgeries, posing a persistent clinical challenge due to the associated chronic pain, organ dysfunction, and heightened healthcare burdens [1,2]. Although numerous commercial anti-adhesion products have been developed, primarily based on natural polymers such as carboxymethyl cellulose, alginate, and hyaluronic acid, or synthetic polymers, including polylactic acid, polyglycolic acid, and polyethylene glycol, these systems remain limited [2,3,4]. Natural polymers offer biocompatibility but are often hampered by inadequate mechanical strength and rapid degradation [5], whereas synthetic polymers provide tunable mechanical properties and stability but may pose biocompatibility risks upon degradation [6]. Moreover, most commercial products act as passive physical barriers without actively modulating the pathological microenvironment that drives adhesion formation, highlighting the need for multifunctional therapeutic strategies.

Postoperative adhesion originates from pathological wound healing following peritoneal injury, characterized by excessive inflammation and reactive oxygen species (ROS) accumulation [2,7,8,9]. This establishes a hypoxic microenvironment that induces oxidative stress, referring to a systemic imbalance between oxidants and antioxidants, which disrupts cellular metabolism and signaling pathways, promoting cell necrosis/apoptosis [9,10,11]. Excessive ROS and reactive nitrogen species amplify the expression of molecules, such as type I collagen, transforming growth factor-β1 (TGF-β1), vascular endothelial growth factor (VEGF), interleukin-6 (IL-6), and tumor necrosis factor-α (TNF-α), which drive aberrant angiogenesis and cellular proliferation despite their physiologic roles in adaptation to hypoxia [12,13,14,15]. Therefore, modulating ROS levels, rather than simply blocking them, is emerging as a central therapeutic strategy to restore balanced healing.

Gelatin, a denatured derivative of collagen, offers extracellular matrix-mimetic properties, biocompatibility, and biodegradability, making it attractive for regenerative applications [16]. However, its clinical translation is often impeded by weak mechanical strength and rapid degradation [5]. To enhance its performance, recent strategies have focused on incorporating polyphenols [17,18,19], such as tannic acid, catechins, or epigallocatechin gallate, which possess strong antioxidant activity, anti-inflammatory effects, and the ability to form dynamic covalent and non-covalent bonds with proteins [20]. In addition, these interactions improve mechanical stability, tissue adhesion, and confer additional bioactive functionality, making gelatin–polyphenol hydrogels attractive candidates for regenerative applications [21,22,23]. Previous studies have highlighted the potential of polyphenol-based hydrogels to scavenge ROS, modulate macrophage polarization, and promote collagen deposition and angiogenesis [24,25], all of which are crucial for wound repair and adhesion prevention. Nevertheless, most existing hydrogels generally exhibit single-function activity, with limited mechanical robustness, short in vivo retention, or insufficient ability in ROS regulation and coordinated immunomodulation under adhesion-prone conditions, thus representing an unresolved challenge.

In this study, we present a multifunctional gelatin–polyphenol hydrogel (GPP20) that integrates rapid gelation, injectability, excellent wet tissue adhesion, self-healing capacity, and ROS scavenging capability. Fabricated via a simple single-step polyphenol engineering strategy, tea polyphenol (TPP) acts simultaneously as a structural reinforcer through dynamic bonding, and as a bioactive modulator via ROS scavenging and macrophage regulation. Unlike conventional hydrogels that serve primarily as passive barriers, GPP20 combines mechanical reinforcement, dynamic antioxidant defense, and immunoregulatory effects within a single platform. In vitro and in vivo evaluations demonstrate that GPP20 effectively prevents tissue adhesion and concurrently promotes wound healing through immunomodulation, angiogenesis, and collagen remodeling. Collectively, these findings highlight GPP20 as a facile and clinically translatable strategy that shifts postoperative therapy from passive adhesion prevention toward an active, multifunctional, and pro-regenerative approach.

## 2. Results and Discussion

### 2.1. Preparation and Characterization of GPP

**Figure 1:**
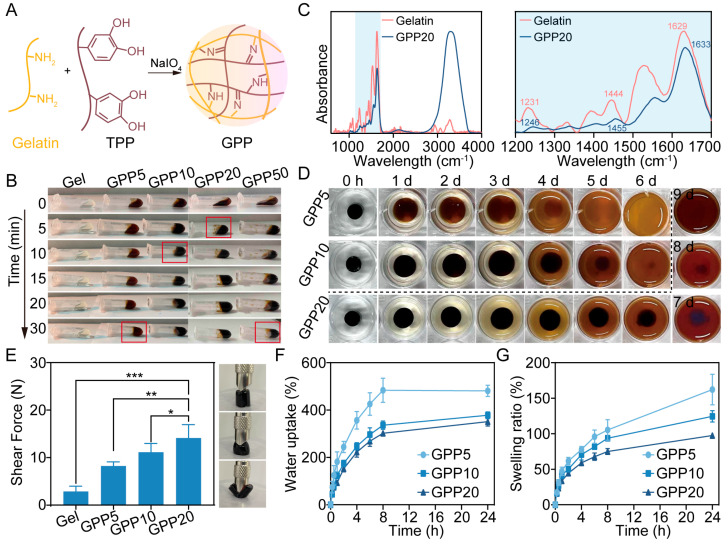
Preparation and characterization of GPP. (**A**) Schematic illustration of the synthetic process for GPP. (**B**) Representative digital images of GPP gelation process with varying TPP concentrations. (**C**) FTIR spectra of GPP20 and pure gelatin. (**D**) Stability of GPP in PBS over time. (**E**) Shear strength of GPP. * *p* < 0.05, ** *p* < 0.01, *** *p* < 0.001. (**F**) Water uptake capacity and (**G**) swelling ratio of GPP.

Gelatin, a denatured collagen derivative, is widely employed as a biomaterial owing to its intrinsic biocompatibility and RGD motifs that facilitate cell–matrix interactions. Under alkaline or neutral conditions, the polyphenolic hydroxyl groups of polyphenol groups are oxidized to reactive quinone groups, which can form covalent bonds with nucleophilic amino acid residues (e.g., lysine and arginine) in gelatin via Schiff base or Michael addition reactions (Figure 1A) [21,26]. Sodium periodate, a strong oxidizer, was used to accelerate polyphenol oxidation and regulate crosslinking kinetics. Gelation time was highly dependent on the TPP concentration, decreasing from 30 min for GPP5 (5% TPP) to 5 min for GPP20 (20% TPP) (Figure 1B). The concentration-dependent acceleration observed aligns with established reaction kinetics principles, wherein higher crosslinker density increases the frequency of molecular collisions and crosslinking events [27]. This rapid gelation profile compares favorably with other catechol-functionalized hydrogel systems, which often require extended setting times exceeding 8~20 min [28], making GPP20 particularly advantageous for in vivo applications where rapid barrier formation is critical to prevent fluid displacement or tissue adhesion.

FTIR analysis further confirmed the formation of these covalent linkages (Figure 1C). Notably, the C=N stretching vibrations from Schiff base formation (1600–1650 cm^−1^) overlapped with the amide I band of gelatin (1629 cm^−1^), resulting in a shifted peak at 1633 cm^−1^ in the GPP spectrum [21,29]. Furthermore, the amide III band (originally at 1231 cm^−1^) shifted to a higher wavenumber (1245 cm^−1^), indicative of C-N bond modification. This observation, coupled with the enhanced intensity and shift to 1455 cm^−1^ of the C-N stretching vibration (previously at 1444 cm^−1^) in the GPP20 spectrum, collectively supports the occurrence of Michael addition reactions between the quinone groups of TPP and the amino groups of gelatin [29,30].

Given that degradability is critical for biomedical applications, the stability of GPP was under physiological conditions (PBS, pH 7.4 and 37 °C). While pure gelatin dissolved rapidly under these conditions, GPP20 exhibited slow degradability over 9 days (Figure 1D), indicating its suitability for applications requiring extended barrier function to prevent postoperative adhesion and support wound healing [31]. Shear strength measurements further confirmed concentration-dependent mechanical reinforcement, ranging from 8.3 N for GPP5 to 14.2 N for GPP20 (Figure 1E). Water absorption was determined via gravimetric analysis, [32] with GPP5, GPP10, and GPP20 exhibiting equilibrium water absorption values of 481%, 378%, and 351% (Figure 1F), and corresponding swelling ratios of 162%, 125%, and 98% (Figure 1G), respectively. The observed enhancements in mechanical strength and the inverse correlation between the TPP concentration and both water absorption and swelling are collectively attributed to the increased crosslinking density in formulations with higher TPP content [27]. This results in a more densely crosslinked network in GPP20, which restricts polymer chain mobility, improves network rigidity, and limits expansion upon hydration, thereby contributing to superior mechanical performance and significantly reduced swelling [33]. Together, these results demonstrate that GPP hydrogels possess strong mechanical properties, low-dimensional swelling, and the ability to maintain structural integrity in aqueous environments, a combination of critical properties for ensuring stable performance and reliable support during tissue regeneration.

### 2.2. Injectability, Tissue Adhesive, and Self-Healing Properties of GPP

**Figure 2:**
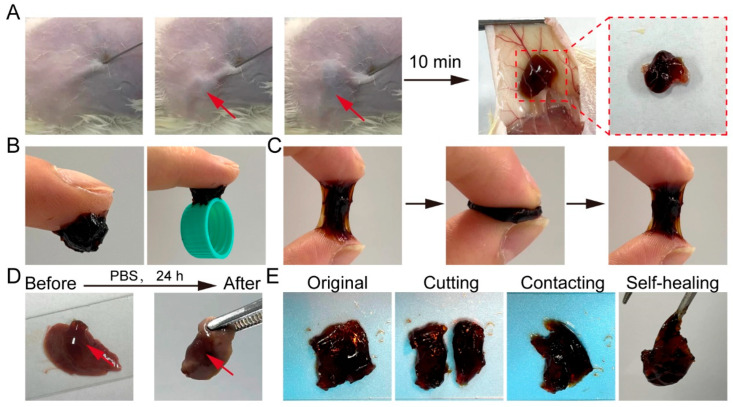
(**A**) In vivo injectability and gelation behavior of GPP via subcutaneous injection. (**B**,**C**) Tissue adhesive of GPP20. (**D**) Long-term tissue adhesion capability of GPP20 after 24 h of immersion in PBS at 37 °C. (**E**) Macroscopic demonstration of the self-healing property of GPP20.

Injectable hydrogels provide significant clinical advantages for repairing irregular defects while minimizing surgical invasiveness. Owing to its rapid gelation and superior mechanical properties, GPP20 was selected for further evaluation. Upon subcutaneous injection into mice, GPP20 formed a stable gel within 10 min (Figure 2A), demonstrating excellent injectability and suitability for in vivo application.

GPP20 exhibited robust tissue adhesion, capable of supporting the weight of a plastic bottle cap (Figure 2B) and maintaining strong adhesive performance after repeated cycles of compression and stretching (Figure 2C). Notably, GPP20 remained firmly adhered to mouse liver tissue after 24 h of immersion in PBS at 37 °C (Figure 2D), confirming its stability under physiological conditions and strong potential for securing internal wounds and effectively isolating the repaired site [34]. The excellent tissue adhesion of GPP is attributed to the abundant catechol groups within the hydrogel network, which form strong interactions with various functional groups (e.g., -NH_2_, -SH, -OH, and -COOH) present on tissue surfaces [21,23,35]. Additionally, bisected fragments of GPP20 rapidly reattached upon gentle contact (Figure 2E), demonstrating an intrinsic self-healing capability, which enables the hydrogel to maintain structural integrity under external mechanical stress, thereby providing durable protection to wound sites [15,36]. Collectively, these properties establish GPP20 as a dynamic and resilient interfacial material capable of preventing postoperative adhesions while supporting wound repair.

### 2.3. Antioxidatant Activity of GPP

**Figure 3:**
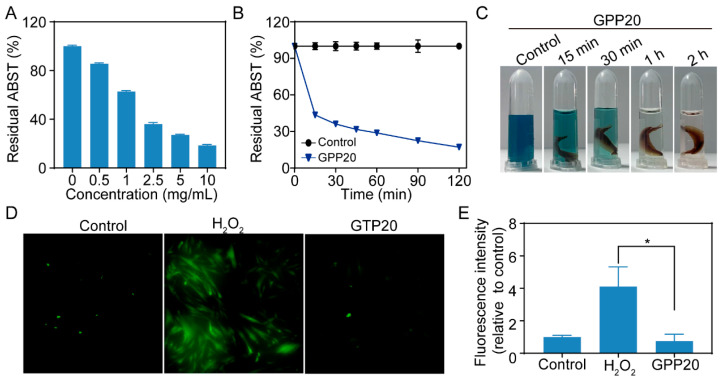
Antioxidant capacity of GPP. (**A**) ABTS scavenging activity of GPP20 at different concentrations. (**B**) Time-dependent ABTS scavenging activity of GPP20. (**C**) Color changes in ABTS solution after incubation with GPP20 for varying times. (**D**) Representative fluorescence images of intracellular reactive oxygen species (ROS) levels across all experimental groups. (**E**) Quantitative analysis of intracellular ROS levels. * *p* < 0.05.

Excessive ROS generated during surgical trauma can damage biomolecules and promote fibrosis, thereby facilitating adhesion formation. Due to its abundant catechol groups, TPP can effectively scavenge free radicals [37,38]. The ABTS^+^· assay revealed that GPP20 exhibited concentration- and time-dependent radical scavenging activity, accompanied by the rapid decolorization of the ABTS solution (Figure 3A–C). To further validate the cellular protective effects of GPP20, mesenchymal stem cells (MSCs) were exposed to a H_2_O_2_-induced oxidative stress environment. DCFH-DA fluorescence analysis demonstrated that GPP20 markedly reduced intracellular ROS accumulation compared with the H_2_O_2_-only group (Figure 3D,E), confirming its protective antioxidant effect at the cellular level.

### 2.4. Biocompatibility of GPP

**Figure 4:**
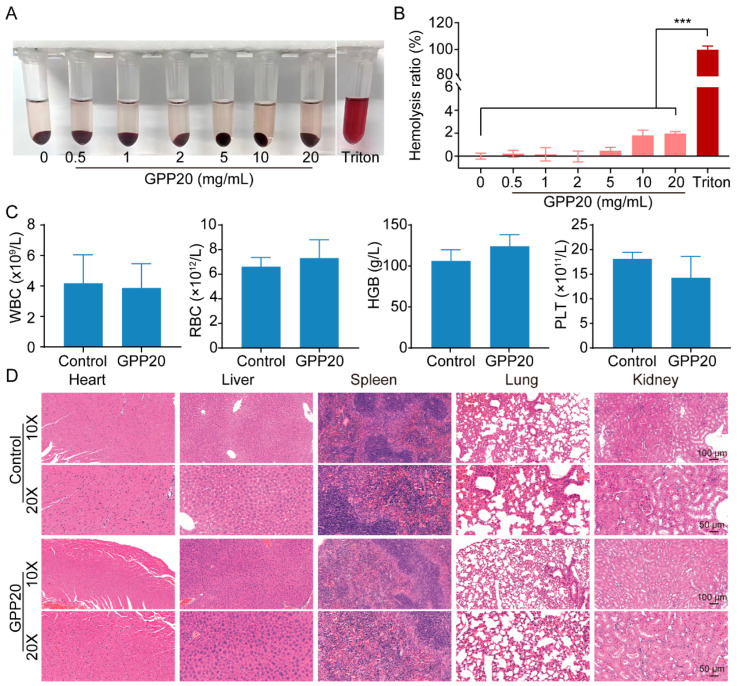
Hemocompatibility of GPP. (**A**) Hemolysis assay of GPP20. Triton (indicated 1% Triton X-100) and PBS were used as the positive control and negative control, respectively. (**B**) Quantification of the hemolysis ratio for GPP20. *** *p* < 0.001. (**C**) Mouse serum blood routine analysis. (**D**) Histological images of the heart, liver, spleen, lung and kidneys of mouse after 4-week implantation of GPP20.

Hemocompatibility is essential for materials in contact with blood [39,40]. Hemolysis assays revealed that GPP20 induced negligible hemolysis compared with PBS group (negative control), whereas 1% Triton X-100 (positive control) caused pronounced hemolysis. Even at 20 mg/mL, the hemolysis ratio of GPP20 remained below 1% (Figure 4A,B), confirming its excellent blood compatibility.

For in vivo safety assessment, GPP20 was implanted subcutaneously in mice. Hematological parameters, including white blood cell (WBC) count, red blood cell (RBC) count, hemoglobin concentration (HGB), hematocrit (HCT), and platelet (PLT) count, showed no significant differences between GPP20-treated and sham groups after 7 days (Figure 4C). H&E staining of major organs (liver, spleen, heart, lung, and kidney) revealed no histological abnormalities (Figure 4D). Collectively, these results demonstrate that GPP20 exhibits excellent systemic and local biocompatibility, supporting its translational potential.

### 2.5. Postoperative Anti-Adhesion Efficacy of GPP

**Figure 5:**
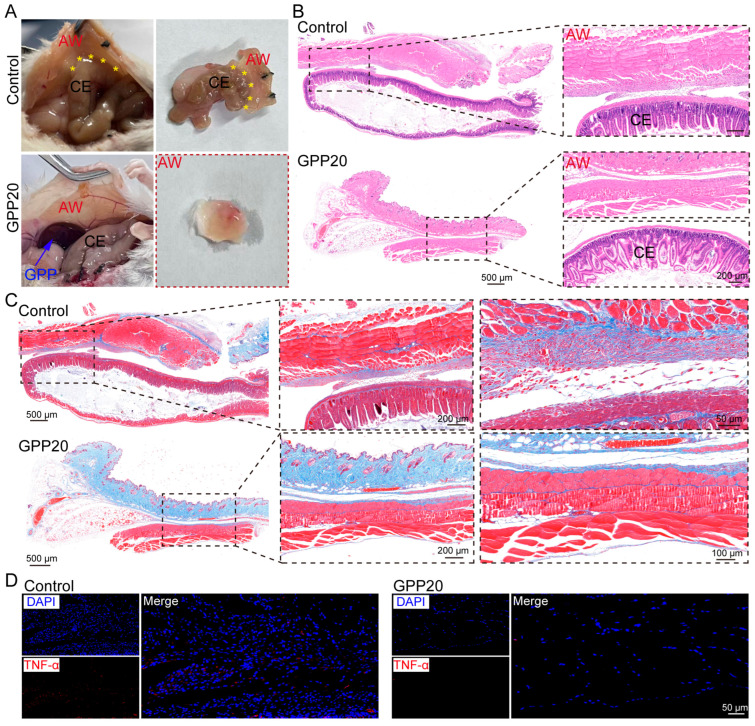
Anti-adhesion effect of GPP in mouse cecal abrasion model. (**A**) Representative pictures of intestinal adhesion treated in GPP20 and control (no treatment) groups. Asterisks (*) indicate adhesion between cecum and abdominal wall. (**B**) H&E staining. CE and AW are short for cecum and abdominal wall, respectively. (**C**) Masson’s trichrome staining. (**D**) TNF-α immunofluorescence staining.

The anti-adhesion efficacy of GPP was systematically assessed in a well-established murine model of cecal abrasion [21]. On day 0, a midline laparotomy was performed to expose the cecum, which was then uniformly abraded using a sterile scalpel until petechial bleeding occurred. The experimental group received a topical application of GPP20 to the injured site, while the control group was treated with sterile saline. At 7 days postoperation, all untreated control mice developed dense, severe adhesions that firmly attached the cecum to the adjacent abdominal wall, resisting separation by gentle traction (Figure 5A). Remarkably, GPP20-treated mice presented with completely clean and free-moving cecal surfaces, showing no macroscopic evidence of adhesion formation.

Histological examination supported these observations. In control animals, H&E staining revealed dense fibrotic tissue bridging the cecum and abdominal wall (Figure 5B). Conversely, tissues from GPP20-treated mice remained clearly separated with intact structures of both the cecum and abdominal wall, indicating the significant anti-adhesive efficacy of GPP20. Furthermore, Masson’s trichrome staining showed substantial collagen deposition within a well-defined adhesive band in the control group on day 7 (Figure 5C), while the GPP20-treated groups showed only minimal, scattered collagen deposition restricted to the subserosal layers without inter-tissue bridging.

The inflammatory response plays a pivotal role in the development of postoperative adhesions. Proinflammatory cytokines such as TNF-α trigger a localized inflammatory imbalance that promotes tissue fibrosis and adhesion formation [14]. Immunofluorescence analysis of adhesion tissues revealed intense TNF-α expression in the control group, indicating a sustained inflammatory microenvironment conducive to adhesion development (Figure 5D). In contrast, GPP20-treated tissues showed markedly reduced TNF-α expression, highlighting the anti-inflammatory effect of the treatment. This suppression of TNF-α is likely mediated by the catechol functional groups in TPP, which confer broad anti-inflammatory properties typical of phenolic compounds. These groups modulate key signaling pathways, including NF-κB, MAPK, and PI3K/Akt, and inhibit the production of inflammatory cytokines such as TNF-α, IL-1β, and IL-6 in macrophages [41,42], thereby disrupting the inflammation–fibrosis cascade and contributing to the anti-adhesive efficacy of GPP20.

### 2.6. Wound Healing Performance of GPP

**Figure 6:**
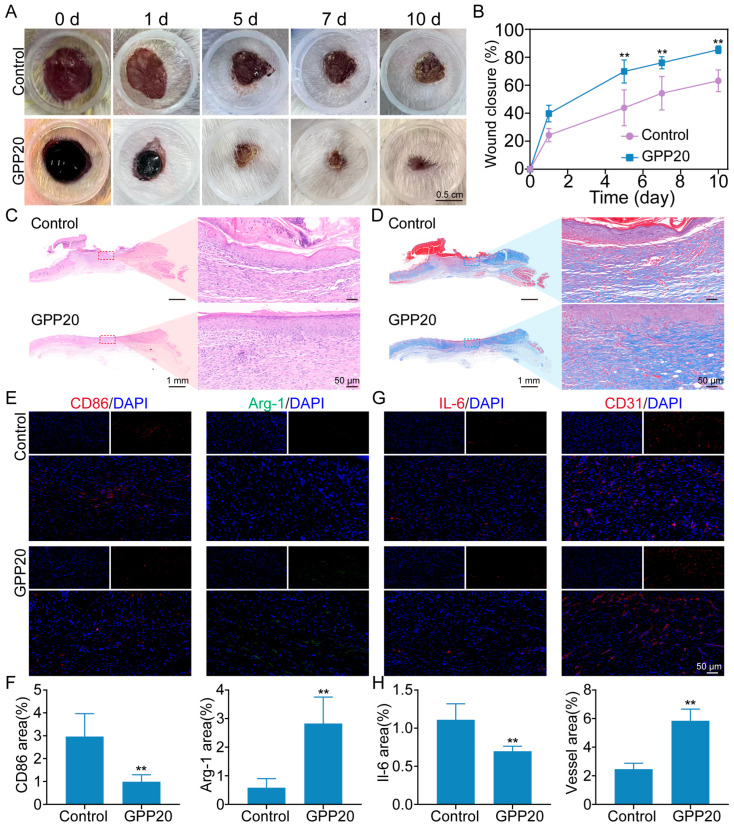
In vivo wound healing evaluation of GPP. (**A**) Representative photographic images of wound healing over time. (**B**) Quantitative analysis of the relative wound area over time. ** *p* < 0.01. (**C**) H&E and (**D**) Masson’s trichrome staining of the skin tissue around wounds. (**E**) Immunofluorescence staining images and (**F**) quantitative analysis of M1 (CD86) and M2 (Arg-1) macrophage markers. ** *p* < 0.01. (**G**) Immunofluorescence staining images and (**H**) quantitative analysis of IL-6 and CD31 expression. ** *p* < 0.01.

The wound healing efficacy of GPP was examined using a full-thickness skin wound model. Skin wounds were treated with GPP20, with an untreated group serving as the control. Figure 6A shows the wound healing process under different treatments on consecutive days. Compared with controls, GPP20-treated wounds showed accelerated closure, reaching 85.3% closure by day 10 versus 65.2% in untreated wounds (Figure 6B).

Histological analysis provided further evidence of enhanced healing. In H&E-stained sections (Figure 6C), the control group exhibited a notable scab on the wound surface and underlying collagen fibers that were loose and irregularly arranged with large gaps. In contrast, the GPP20-treated group showed a relatively smooth and intact epithelial surface with more dense and orderly arranged collagen fibers. This improved collagen deposition and organization was further confirmed by Masson’s trichrome staining (Figure 6D), which revealed a more structured and abundant collagen matrix (blue) in the GPP20 group compared to the disorganized and sparse collagen network in the control group, indicating advanced tissue remodeling.

To elucidate the underlying mechanism, macrophage polarization was assessed [43]. Macrophages are one of the key early inflammatory mediators. Typically, macrophages first adopt a proinflammatory type (M1) during wound healing to secrete proinflammatory and chemotactic factors, and then switch to an anti-inflammatory type (M2) phenotype to facilitate tissue repair and regeneration. We evaluated the macrophage phenotype during the healing using immunofluorescent staining (Figure 6E,F). On day 10, M1 and M2 macrophages in all groups were stained with CD86 and Arginase-1 (Arg-1), respectively [43]. The positive area of the M1 marker CD86 in the GPP20 group (1.0 ± 0.3%) was significantly lower than that of in the control (3.0 ± 1.0%) group, and the positive area of the M2 marker Arg-1 in the GPP20 (2.8 ± 0.9%) was significantly higher than that in the control (0.6 ± 0.3%). In addition, we further investigated the inflammation response and angiogenesis on day 10 through immunofluorescence staining (Figure 6G,H). The results showed that the interleukin-6 (IL-6) expression was markedly reduced in the GPP20 group (0.7 ± 0.1%) compared with the control group (1.1 ± 0.2%). Additionally, platelet endothelial cell adhesion molecule-1 (CD31) staining revealed a higher angiogenesis density in the GPP20 group (5.8 ± 0.8%) than in the control group (2.5 ± 0.4%). These results demonstrate that GPP20 not only accelerates wound closure but also orchestrates a favorable immune microenvironment and vascular remodeling, thereby supporting efficient tissue repair.

As a physical barrier, an ideal anti-adhesion material should possess excellent biocompatibility, suitable retention time, an appropriate degradation rate, and the ability to promote wound healing. However, existing polymeric materials still fall short in meeting these comprehensive clinical demands (Table S1, Supporting Information). For instance, gelatin-based systems despite offering antioxidant/anti-inflammatory functions, hemostasis, and wet-adhesion, suffered from complex multi-step preparation, compositional heterogeneity, and lack of injectability [6,7]. Human amniotic membrane-derived materials supported wound healing but involved complicated fabrication and degraded too rapidly in vivo [44]. Microgel-based cream hydrogels formed by the conjugation reaction of epigallocatechin-3-gallate (EGCG) and hyaluronic acid (HA)-based microgels with poly(vinyl alcohol) (PVA), showed injectability and anti-inflammatory activity, and possessed prolonged degradation (>14 d), but lacked evidence of wet adhesion and hemostasis functions [24]. Other synthetic hydrogels also face significant drawbacks. Polysaccharide hemoadhican–polyethylene glycol diglycidyl ether (PEGDE) hydrogels could be prepared in one step but degraded over an excessively long period (~42 days) and exhibited functional monotony due to random crosslinking, which eliminated the structural heterogeneity [45]. Dopamine-modified oxidized-HA/polyurethane hydrogels provided moderate stability (~7 days) but relied on two-step synthesis and have not been verified for hemostasis capacity [14]. Although carboxymethyl chitosan/PEG hydrogels possessed multiple advantages, including injectability, two-step preparation, anti-inflammatory activity, wet adhesion, self-healing, and suitable degradability (<2 weeks), their hemostasis and wound healing capability have not been confirmed [31]. An integrally formed Janus hydrogel exhibited strong wet tissue adhesion and anti-postoperative adhesion properties along with facile one-step preparation, rendering it promising for internal wound repair; however, it was not injectable [34].

Collectively, these strategies are often hindered by multi-step fabrication, compositional complexity, inadequate functional integration, or mismatched degradation kinetics relative to the clinical adhesion risk window. In contrast, GPP20 developed in this work overcomes these hurdles through a uniquely convergent design: it combines straightforward one-step preparation with exceptional functional performance. Key advantages include rapid gelation (within 5 min), injectability, sustained wet adhesion (>24 h), and autonomous self-healing, supported by robust mechanical properties (shear stress of 14.2 N). Beyond its physical barrier function, GPP20 demonstrates pronounced bioactivity, effectively scavenging ROS (82% ABTS radical scavenging efficiency), modulating immune response (M1 to M2 macrophage polarization), accelerating hemostasis, and promoting angiogenesis, collectively leading to efficient wound closure (85% in 10 days). Its programmed biodegradation (~9 days) aligns with the clinical adhesion risk window, further mitigating fibrotic adhesion through coordinated inflammation resolution and collagen remodeling.

## 3. Conclusions

In summary, GPP20, in a single easily fabricated system, integrates the essential attributes of injectability, adhesion, antioxidative capacity, immunomodulation, and regenerative support, which have been hitherto achieved only separately in previous platforms. This comprehensive functionality positions GPP20 as a unified, clinically viable strategy for simultaneous anti-adhesion treatment and wound recovery.

## 4. Materials and Methods

Materials: Gelatin and TPP (total polyphenol content > 98%) were purchased from Shanghai Aladdin Biochemical Technology Co., Ltd. (Shanghai, China). DPPH was purchased from Macklin (Shanghai, China). ABTS was purchased from Kaiwei Chemical (Shanghai, China). Phosphate-buffered saline (PBS) and trypsin were purchased from KeyGen (Nanjing, China).

Preparation of GPP: To fabricate GPP, a certain amount of TPP was added to the gelatin solution (15% *w/v* in ddH_2_O) and vortexed (500 rpm) for 10 min at 37 °C to ensure uniform mixing. The hydrogel formulations containing varying TPP concentrations (0%, 5%, 10%, and 20% *w*/*w* of gelatin) were prepared and designated as Gel, GPP5, GPP10, GPP20, and GPP50, respectively. Subsequently, sodium periodate was added to the mixture, and the pH was adjusted to 7–8 using sodium hydroxide (NaOH). The resulting precursor solution was then placed in a 37 °C incubator for gelation.

Injection test: The pre-gel solution was loaded into a 1 mL syringe and subcutaneously injected into the dorsal region of mice using a 26-gauge needle. The formation and stability of the hydrogel were monitored 10 min post-injection, at which point a stable gel had formed in situ.

Stability: Prior to testing, the hydrogels were incubated in PBS (pH 7.4) at 37 °C in a 12-well plate (with each well containing 2 mL of PBS). The stability of GPP was evaluated daily by carefully observing and imaging the extent of fragmentation and degradation of the hydrogels. Photographs were taken for record keeping.

Swelling ability: Prior to testing, the hydrogel (*n* = 4 per group) was pre-weighed and immersed in a copious amount of PBS (pH 7.4) at 37 °C. At defined time intervals (0 min, 15 min, 30 min, 1 h, 2 h, 4 h, 6 h, 8 h, and 24 h), each hydrogel was carefully removed from the PBS bath, blotted gently with filter paper to remove excess surface water, weighed, and returned to the PBS bath to continue swelling. The swelling ratio was calculated as [46]

Water uptake (%) = (W_t_ − W_0_)/W_0_ × 100%, (1)

Swelling ratio (%) = (L_t_ − L_0_)/L_0_ × 100%, (2)

where W_t_ and W_0_ represent the hydrogel weight at time t and initial weight, respectively. L_t_ and L_0_ represent the corresponding hydrogel lengths.

ABTS scavenging ability: The ABTS assay was used to determine the radical scavenging activity of the hydrogel [47]. ABTS solution and potassium persulfate were mixed at a 1:1 ratio and incubated in the dark for 12 h. The solution was then diluted to an absorbance of 0.70  ±  0.05 at 734 nm in PBS. Hydrogels were incubated with the ABTS working solution for 1 h in the dark at 37 °C, with blank ABTS solution as control. The OD value at 734 nm was measured. The scavenging efficiency was calculated as follows:

ABTS scavenging effect (%) = [(OD_Con_ − OD_GPP_)/OD_Con_] × 100%. (3)

Cellular ROS scavenging activity: MSCs (Procell, Wuhan, China) were cultured in DMEM+GlutaMAX medium (Gibco, New York, NY, USA), supplemented with 10% (*v*/*v*) FBS and 1% (*w*/*v*) penicillin/streptomycin (Yeasen, Shanghai, China), in a humidified atmosphere containing 5% CO_2_ at 37 °C. After reaching ∼80% confluence, the cells were exposed to H_2_O_2_ (100 μM) in the presence or absence of GPP20. For ROS detection, cells were incubated with DCFH-DA for 20 min in the dark, followed by fluorescence microscopy imaging.

Hemolysis assay: Fresh anticoagulant whole blood from mice was centrifuged and repeatedly washed with PBS to obtain red blood cells. The hydrogel was added to the diluted red cells (8% *v*/*v*) and incubated at 37 °C. Triton X-100 (1%) and PBS were used as positive and negative controls, respectively. After incubation, the samples were centrifuged at 3000 rpm for 15 min, and the absorbance of the supernatant was measured at 540 nm using a Bio-Tek SYNERGY2 microplate reader (Bio-Tek Instruments, Inc., Winooski, VT, USA). The hemolysis rate was calculated as follows:

Hemolysis rate(%) = (OD_GPP_ − OD_negative_)/(OD_positive_ − OD_negative_) × 100%. (4)

In vivo anti-adhesion evaluation: All animal procedures were performed in compliance with the guidelines approved by the animal research committee of Yangzhou University (SYXK(Su) 2022-0044). A mouse cecal abrasion model was established to evaluate anti-adhesion performance of GPP [21]. Briefly, after hair removal, a midline abdominal incision (3–4 cm in length) was made using surgical scissors to open the peritoneal cavity and expose the cecum. The serosal surface of the cecum was gently abraded with a sterile scalpel until petechial hemorrhage occurred. Then, a volume of 0.2 mL of the pre-gel GPP20 solution was applied to the wounded cecal surface and allowed to crosslink for 5 min, forming a hydrogel with an average thickness of 2 mm. In the control group, the injured cecum was treated with an equal volume of sterile saline. The abdominal wall was closed in two layers using 3/0 silk sutures. Seven days after surgery, the peritoneal cavity was reopened to assess adhesion formation between the cecum and the abdominal wall. Tissue samples from the injury and adhesion sites were harvested for further histological evaluation, including by H&E staining, Masson’s trichrome staining, and immunofluorescence analysis.

Wound healing: Mice were fed for 1 week before operation to adapt to the environment. After anesthetization and depilation, a diameter of 8 mm full-thickness excisional wound was created on the dorsal skin. A volume of 0.5 mL of the pre-gel GPP20 solution was applied via injection to cover the entire wound area, where it formed a stable hydrogel in situ within approximately 5 min, forming a hydrogel with an average thickness of 4 mm. No further re-dressing was performed throughout the healing process. The untreated group was used as negative control. The wounds were photographed and the wound closure area was calculated by Image Pro Plus 6. The wound healing ratio (%) was calculated using the following equation: wound contractions = (A_0_ − A_t_) /A_0_ × 100%. The wound area on day 0 and day t was labeled as A_0_ and A_t_, respectively. On day 10, the healed skins were collected for H&E staining, Masson’s trichrome staining, and immunofluorescence staining.

Statistical analysis: The analysis data are expressed as the mean ± SD deviation of at least triplicate samples. GraphPad Prism 8 was used for one-way or two-way ANOVA to obtain the significant difference. When * *p* < 0.05, ** *p* < 0.01, and *** *p* < 0.001, the significant differences between groups were marked.

## Data Availability

The original contributions presented in the study are included in the article; further inquiries can be directed to the corresponding author.

## Supplementary Materials

The following supporting information can be downloaded at: https://www.mdpi.com/article/10.3390/gels11100797/s1, Table S1: Comprehensive evaluation of materials for preventing postoperative adhesion.

## Abbreviations

The following abbreviations are used in this manuscript:

ROS	Reactive oxygen species
VEGF	Vascular endothelial growth factor
IL-6	Interleukin-6
TNF-α	Tumor necrosis factor-α
PBS	Phosphate-buffered saline
