# Supplementary material for: Single-Step Engineered Gelatin-Based Hydrogel for Integrated Prevention of Postoperative Adhesion and Promotion of Wound Healing
Source: Gels. 2025 Oct 2;11(10):797. doi: 10.3390/gels11100797 (PMC12562426; doi:10.3390/gels11100797)
Supplement: Supplementary file 1 [file gels-11-00797-s001.zip › gels-3881396-supplementary.pdf]

Supporting Information

Single-Step Engineered Gelatin-Based Hydrogel for Integrated Prevention of Postoperative Adhesion and Promotion of Wound Healing

Xinyu Wu[1,#], Lei Sun[2,#], Jianmei Chen[1], Meiling Su[1], Zongguang Liu[3,*]

[1] Key Laboratory of the Jiangsu Higher Education Institutions for Integrated Traditional Chinese and Western Medicine in Senile Diseases Control, School of Traditional Chinese Medicine, Faculty of Medicine, Yangzhou University, Yangzhou, 225009, P.R. China. cjm@yzu.edu.cn (J.C.)

[2] School of Pharmacy, East China Normal University, Shanghai 200062, P.R. China. ylyh_sl@163.com (L.S.)

[3] Microelectronics Industry Research Institute, College of Physics Science and Technology, Yangzhou University, Yangzhou, 225009, P.R. China

* Correspondence: zgliu@yzu.edu.cn (Z.L.)

[#] These authors contributed equally to this work.

Table S1. Comprehensive evaluation of materials for preventing postoperative adhesion.

Material		Preparation	Injectability	Antioxidant Antiinflammatory	Wet adhesion	Self-healing	Hemostasis	Degradability (*in vivo*)	Wound-healing capability
[1]gelatin/GelMA/catechol-modified oxidized HA/PCL	Membrane	Multi-step	×	√	√	not mentioned	√	~2 weeks	√
[2]GelMA/gelatin/p(AA-co-NHSMA)/PLA/p(MPC-co-MBP)	Patch	Multi-step	×	√	√	×	√	~4 weeks	potential
[3]human amniotic membrane-ECM/MSCs-secretome	Hydrogel	Multi-step	√	not mentioned	not mentioned	not mentioned	√	3-8 d	√
[4]PBA/EGCG/HA/DMTMM/PVA	Hydrogel	Multi-step	√	√	not mentioned	√	not mentioned	>14 d	not mentioned
[5]polysaccharide hemoadhican/PEGDE	Hydrogel	One-step	√	√	not mentioned	√	potential	42 d	potential
[6]dopamine-modified oxidized HA/dihydrazide-terminated polyurethane	Hydrogel	Two-step	√	√	not mentioned	√	not mentioned	~7 d	potential
[7]carboxymethyl chitosan/dialdehyde-PEG	Hydrogel	Two-step	√	√	√	√	not mentioned	<2 weeks	not mentioned
[8]CTAB/DMAPS/AA/LMA	Hydrogel	One-step	×	√	√	not mentioned	not mentioned	>14 d	√
**GPP20 (gelatin/tea polyphenol) (this work)**	Hydrogel	One-step	√	√	√	√	√	>7 d	√

HA: hyaluronic acid; PCL: poly(ϵ-caprolactone); PEG: polyethylene glycol; PEGDE: polyethylene glycol diglycidyl ether; DMAPS: [2-(methacryloyloxy) ethyl] dimethyl-(3-sulfopropyl) ammonium hydroxide; LMA: lauryl methacrylate; CTAB: hexadecyl trimethyl ammonium bromide; GelMA: Gelatin methacrylate; p(AA-co-NHSMA): poly(acrylic acid)-co-poly(methacrylic acid N-hydroxysuccinimide ester); PLA: polylactic acid.

References

1. Y. Lv, F. Cai, X. Zhao, X. Zhu, F. Wei, Y. Zheng, X. Shi, J. Yang. Bioinspired microstructured janus bioadhesive for the prevention of abdominal and intrauterine adhesions, *Adv. Funct. Mater.* **2024**, *34*, 2314402.

2. W. Peng, C. Liu, Y. Lai, Y. Wang, P. Liu, J. Shen. An adhesive/anti-adhesive janus tissue patch for efficient closure of bleeding tissue with inhibited postoperative adhesion, *Adv. Sci.* **2023**, e2301427.

3. H. Zhu, F. Liu, Y. Liao, H. Li, K. Gao, X. Liang, H. Jiang, F. Chen, J. Wu, Q. Wang, Y. Wang, X. Shuai, X. Yi. Biomimetic nanostructural materials based on placental amniotic membrane-derived nanofibers for self-healing and anti-adhesion during cesarean section, *Biomaterials* **2025**, *317*, 123081.

4. B. Liu, Y. Kong, O. A. Alimi, M. A. Kuss, H. Tu, W. Hu, A. Rafay, K. Vikas, W. Shi, M. Lerner, W. L. Berry, Y. Li, M. A. Carlson, B. Duan. Multifunctional microgel-based cream hydrogels for postoperative abdominal adhesion prevention, *ACS Nano* **2023**, *17*, 3847-3864.

5. W. Lu, X. Wang, C. Kong, S. Chen, C. Hu, J. Zhang. Hemoadhican-based bioabsorbable hydrogel for preventing postoperative adhesions, *ACS Appl. Mater. Interfaces* **2024**, *16*, 17267-17284.

6. L. Yuan, H. Wei, Z. Pan, X. Deng, L. Yang, Y. Wang, D. Lu, Z. Li, F. Luo, J. Li, H. Tan. A bioinspired injectable antioxidant hydrogel for prevention of postoperative adhesion, *J. Mater. Chem. B* **2024**, *12*, 6968-6980.

7. M. Wang, S. Lin, M. Liu, J. Jiao, H. Mi, J. Sun, Y. Liu, R. Guo, S. Liu, H. Fu, Y. Yang, R. Li. An injectable and rapidly degraded carboxymethyl chitosan/polyethylene glycol hydrogel for postoperative antiadhesion, *Chem. Eng. J.* **2023**, *463*, 142283.

8. H. Wang, X. Yi, T. Liu, J. Liu, Q. Wu, Y. Ding, Z. Liu, Q. Wang. An integrally formed janus hydrogel for robust wet-tissue adhesive and anti-postoperative adhesion, *Adv. Mater.* **2023**, e2300394.
